# Durability of the Major Determinants of Endurance Performance Is Not Different After 15 kJ·kg^−1^ of Moderate or Heavy Exercise

**DOI:** 10.1002/ejsc.70039

**Published:** 2025-08-15

**Authors:** George Evans, Alan Chorley, Jamie Highton

**Affiliations:** ^1^ Division of Public Health Sport and Wellbeing University of Chester Chester UK; ^2^ School of Life and Medical Sciences University of Hertfordshire Hatfield UK

**Keywords:** cardiovascular/cardiorespiratory, exercise, fatigue, performance, physiology

## Abstract

Durability, or resilience to deteriorations in physiological endurance characteristics during exercise, is posited to have important implications for endurance performance. However, little is known about the effects of exercise intensity on the durability of important endurance parameters. The aim of this study was to compare changes in peak ramp power (power achieved at the end of a ramp test), $\dot{V}$O_2max_, lactate threshold, critical power, gross efficiency and W′ after work‐matched moderate and heavy exercise bouts. Twelve competitive cyclists ($\dot{V}$O_2max_ = 62.1 ± 4.4 mL·kg^−1^ min^−1^) performed exercise testing before and after completing 15 kJ·kg^−1^ work bouts in the moderate (duration = 4837 ± 675 s) and heavy (4000 ± 537 s) exercise domain. Significant declines were seen in peak ramp power (baseline = 412.6 ± 64.5 W, moderate = 380.2 ± 59.7 W and heavy = 374.8 ± 59 W) and W’ (baseline = 7.8 ± 4 kJ, moderate = 4 ± 3.6 kJ and heavy = 3.6 ± 2.4 kJ)—all other parameters did not change. There were no significant differences in the magnitude of decline between the moderate and heavy work bouts for any variable. For the first time, we show that durability of important determinants of endurance performance is not different when 15 kJ·kg^−1^ of moderate or heavy work is performed, suggesting that exercise domain does not influence durability for exercise lasting ∼60–90 min. Further research is needed to explore exercise of longer durations and associated physiological mechanisms for deteriorations in relevant parameters.

## Highlights

- This is the first study to explore laboratory measured changes in all the primary physiological determinants of endurance cycling (i.e., durability) after work‐matched prior exercise in the moderate and heavy domains.
- We found that the exercise domain did not affect durability of peak ramp power, $\dot{V}$O_2max_, lactate threshold, economy, critical power or W’. Peak ramp power and W′ were the only variables to significantly decline after prior exercise.
- Coaches and practitioners should be aware that as little as 15 kJ·kg^−1^ work in the moderate and heavy exercise domain can reduce peak ramp power and W′, which could affect performance and training intensity.

## 1 Introduction

Endurance performance is often modelled using maximal oxygen uptake ($\dot{V}$O_2max_), the maximal sustainable fraction of $\dot{V}$O_2max_ (performance $\dot{V}$O_2_, frequently indicated by the lactate or gas‐exchange threshold) and exercise economy (Coyle 1999; Jones et al. 2021; Joyner 1991; Midgley et al. 2007). These three physiological parameters (‘the big 3’) can explain 72%–88% of the between‐subject variance in long distance running (Di Prampero et al. 1986; Jones et al. 2021, 2019; Joyner 1991) and cycling (Maunder et al. 2022) performance. A related paradigm is the hyperbolic relationship between power and the duration it can be sustained, from which critical power (CP; the boundary between heavy and severe exercise intensity domains or steady‐state and nonsteady‐state exercise) and W’ (a fixed amount of work that can be performed above the CP) can be derived. These parameters can explain 79% of the variation in performance of a 16.1 km TT (Morgan et al. 2019).

Laboratory testing of physiological markers, such as CP, W′, $\dot{V}$O_2max_ (and its associated speed/power), exercise economy and lactate threshold, usually occurs when athletes are in a well‐rested state (Maunder et al. 2021). Interestingly, recent research has demonstrated that these variables may decline during prolonged exercise (Clark et al. 2018, 2019a, 2019b; Spragg et al. 2023; Stevenson et al. 2022). The ability to avoid or minimise this decline has been termed an athlete's durability; hereby defined as an individual's resilience to deteriorations in physiological profiling characteristics during prolonged exercise (Jones 2023; Maunder et al. 2021; Spragg et al. 2022). Durability is likely instrumental for athletes competing in endurance events, such as road cycling, who must produce high power outputs after several hours of exercise (Sanders et al. 2019). Indeed, durability has recently been suggested to be the ‘fourth parameter’ of endurance performance (Jones 2023; Spragg et al. 2022)

Given their relationship with endurance performance, it would seem prudent to assess the durability of CP, W’ $\dot{V}$O_2max_, LT and economy as part of endurance athlete profiling. Owing to relatively recent advancements in testing protocols (Burnley et al. 2006; Chorley et al. 2020; Chorley and Lamb 2020; Murgatroyd et al. 2014; Vanhatalo et al. 2008), the durability of these parameters could feasibly be assessed in a single visit (Burnley et al. 2006; Chorley and Lamb 2020; Murgatroyd et al. 2014; Vanhatalo et al. 2008). However, to the best of our knowledge, no study has attempted to assess the durability of *all* parameters in the same participants.

When comparing physiological parameters between a fresh and fatigued state, it is also important to consider the characteristics of the fatiguing protocol. The ‘task‐dependency’ of fatigue is well‐known (Enoka and Duchateau 2008), and different exercise intensity domains are associated with distinct fatigue mechanisms (Burnley and Jones 2018). Therefore, it is likely that any deterioration in endurance parameters is associated with the nature of the exercise preceding it. Understandably, initial investigations into durability used exercise performed in a single domain. These studies demonstrated reductions in some endurance parameters after 2 h of heavy (CP and W’; Clark et al. 2018, 2019a, 2019b) and moderate (power output at the ventilatory threshold; Stevenson et al. 2022) exercise. Subsequently, recent field‐based studies have shown greater reductions in some, but not all, endurance parameters after high intensity exercise. In elite cyclists, Spragg et al. (2024) observed mean power was reduced during a 15 s and 3 min test to a greater extent after 2000 kJ of work performed below CP compared to less work performed repeatedly above CP (5 × 8 min at 105%–110%). Power during a 12 min test and CP were not affected. Two similar studies have compared mean power during different duration performance tests after work‐matched bouts of exercise below and above CP. Barranco‐Gil et al. (2024) observed a greater reduction in W′ and mean power during a 2 min test after 15 kJ·kg^−1^ work performed above CP (compared to the same amount of work performed below CP). Power during 5 and 12 min tests and CP were not different. In contrast, Mateo‐March et al. (2024) reported significant reductions in CP and power during 5 s, 5, 10 and 20 min tests after work‐matched exercise above CP, but not below.

It is notable that all studies comparing the effects of exercise intensity on durability have (a) been field‐based, (b) not measured all the parameters associated with endurance performance (i.e., $\dot{V}$O_2max_, sustainable fraction of $\dot{V}$O_2max_, efficiency, CP and W′) and (c) have compared work bouts performed below and above CP. Controlled laboratory investigations of all important endurance parameters after exercise in the moderate and heavy domain are therefore warranted. Accordingly, the aim of the present study was to examine laboratory‐measured changes in peak ramp power, $\dot{V}$O_2max_, LT, gross efficiency, CP and W′ after 15 kJ·kg^−1^ work performed in the moderate and heavy domain.

## 2 Methods

### 2.1 Participants

Twelve (9 male and 3 female) competitive cyclists participated in the study; characteristics are presented in Table 1. To meet selection criteria, participants were required to be between the ages of 18 and 50 years and be at a level equivalent to, or better than, British Cycling third category (British Cycling racing licence categories: Elite, first, second, third and fourth). Therefore, all participants had regularly competed and scored points in competitive cycling road races. A‐priori sample size estimation (G*power, version 3.1.9.7; Universität Düsseldorf, Germany) indicated a total sample size of 11 was required to detect the magnitude of changes in critical power associated with fatigue (260 ± 37 W vs. 236 ± 47 W) seen by Clark et al. (2019b), and a sample as small as 7 would be sufficiently powerful to detect differences in critical power after 7.5 kJ^‐^kg^−1^ of heavy and severe exercise according to Mateo‐March et al. (2024).

**TABLE 1:** Participant baseline characteristics.

Characteristic	Mean ± SD
Age (years)	31.4 ± 9.2
Stature (cm)	180.3 ± 11.9
Mass (kg)	73.3 ± 12.9
^a^Mean weekly training volume (h)	9.3 ± 3.1
Lactate threshold (W)	255 ± 46
Gross efficiency (%)	21.8 ± 0.9
$\dot{V}$O_2max_ (ml·kg^−1^ min^−1^)	62.1 ± 4.4
Peak ramp power (W)	413 ± 64.5
Critical power (W)	343 ± 68.3
W’‐prime (J)	8210 ± 4018

^a^ 6 participants provided their weekly training volume (h).

Participants completed an informed consent form, pretest health questionnaire and had blood pressure and resting heart rate (HR) measured before commencing any exercise. The study was approved by the Faculty of Medicine and Life Sciences Research Ethics Committee at the University of Chester (Approval no. 1977‐23‐GE‐SES).

### 2.2 Experimental Design

Participants completed three laboratory visits separated by at least two days over a period of 23 ± 16 days. Visit one involved a ramp all‐out protocol to determine participants' baseline lactate threshold, gross efficiency, $\dot{V}$O_2max_, peak ramp power, CP and W’. Then, in a randomised counterbalanced order, visits two and three required participants to perform 15 kJ·kg^−1^ of moderate or heavy intensity cycling before completing another ramp all‐out protocol. All testing was performed at the same time of day (± 1.5 h) in an air‐conditioned laboratory set to 20°C. Participants were instructed to consume a similar amount of carbohydrates and avoid taking any nonhabitual nutritional supplements 24 h prior to each visit. All participants kept a food‐diary (MyFitnessPal) in the 24 h leading up to the visit and verbally agreed to keep energy and carbohydrate intake similar during the 24 h leading up to the following visits. They were also asked to refrain from undertaking any severe exercise 48 h before each visit.

### 2.3 Procedures

**FIGURE 1:**
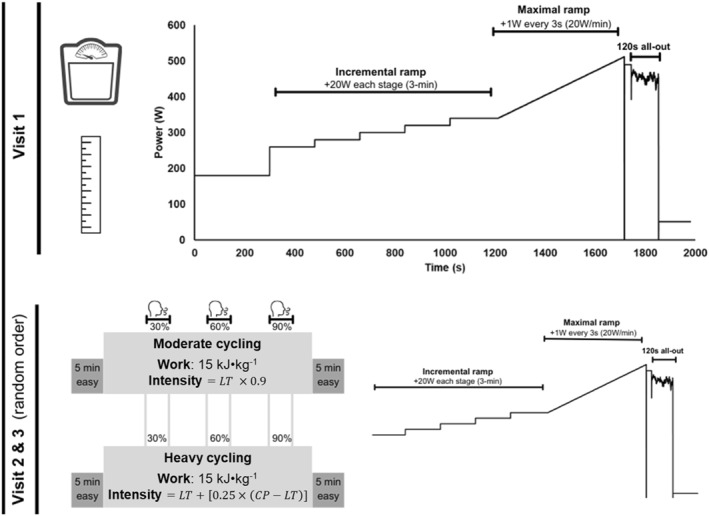
Schematic of study design.

During the initial visit, participants' height (cm) and body mass (kg) were recorded. Resting values of capillary blood lactate were also measured with a portable blood lactate analyser (Lactate Pro 2, Arkray, Kyoto, Japan) and a facemask for a portable online gas analyser (Cosmed K5) was fitted. Then, a ramp all‐out test (Figure 1) to determine lactate threshold, $\dot{V}$O_2max_, gross efficiency, peak ramp power, CP and W′ was performed on an electronically braked ergometer (Lode Excalibur Sport, Lode BV, Groningen, Netherlands). The ramp all‐out protocol began with participants cycling at a self‐determined ‘easy’ pace (140–180 W) for five minutes before commencing an incremental ramp test (+ 20 W every 3 min) to determine LT and gross efficiency, with starting power output of the incremental ramp test adjusted for each participant using recent training data. Rating of perceived exertion (RPE) and blood lactate was sampled in the last 30 s of each stage; LT was determined as the stage before blood lactate values exceeded 1 mmol·L^−1^ above resting values (Bentley et al. 2007). Gross efficiency was calculated during the last 60 s of the stage prior to attaining LT. Once a > 1 mmol·L^−1^ rise in blood lactate was observed, a maximal ramp test to exhaustion (20 W·min^−1^) commenced from the power output at which the rise in lactate occurred. RPE was taken every minute until the participants' cadence fell below 50 rpm; this was indicative of volitional exhaustion and therefore peak ramp power. At this point, the resistance of the ergometer was removed momentarily (1 s), allowing participants to regain a suitable cadence, before a load > 30 W above predicted CP was added to ensure a more comprehensive depletion of W’ (Chorley et al. 2020). Participants cycled at this load until volitional exhaustion (< 50 rpm) occurred once more; the ergometer then automatically changed from hyperbolic mode to a linear factor resistance, and participants were instructed to cycle at an all‐out intensity for 120 s. Power output and time remaining were withheld during this stage to limit pacing. The mean power during the last 30 s of this stage was used to calculate CP (Murgatroyd et al. 2014; Chorley et al. 2020) unless there was evidence of pacing (last 30 s > 10 W higher than the last 60 s average), in which case, the sampling timeframe was extended. The total work accumulated above CP during the maximal ramp test and the load > 30 W above CP prior to the 120 s all‐out was calculated to be W’. The highest mean $\dot{V}$O_2_ (ml·kg^−1^ min^−1^) for 30 s from breath‐by‐breath analysis (without errant breaths) was deemed to be $\dot{V}$O_2max_. The linear factor resistance (*α*) of the ergometer during the 120 s all‐out phase was calculated so that estimated CP would be achieved at the participants' preferred cadence:

(1) $$\text{Linear}\;\text{factor}\;\left(\alpha \right)=\frac{\text{Power}\;\text{output}\;\text{at}\;\text{estimated}\;\text{CP}}{{\text{Preferred}\;\text{cadence}}^{2}},$$

where estimates of CP were based on previous CP testing or maximal mean power observed from training/race data (Chorley et al. 2020).

During the following visits, the same testing procedure (ramp all‐out) was performed 5 min after accumulating 15 kJ·kg^−1^ of either moderate or heavy intensity cycling (Figure 1) on the same electronically braked ergometer. This amount of work was selected as it has been shown to induce reductions in mean maximal power, critical power and W’ (Barranco‐Gil et al. 2024; Mateo‐March et al. 2022, 2024). Before the moderate and heavy domain exercise bouts commenced, participants completed 5 min at an easy pace (140–180 W). The intensity of the moderate bout was fixed at 90% of the power output at the LT established in the preliminary testing (Stevenson et al. 2022). The intensity of the heavy bout was fixed at LT + 25% of the difference between LT and CP (Clark et al. 2018). Collection of expired gasses was taken intermittently (for 3 min at: 30%, 60% and 90% of the 15 kJ·kg^−1^) during these work‐bouts to confirm that participants were in the correct exercise domain. Once the moderate or heavy bout was completed, a 5 min period at an easy pace (140–180 W) began before participants started the same ramp all‐out test procedure that was completed in the initial visit (Figure 1).

### 2.4 Data Analysis

Descriptive statistics (mean ± SD) were calculated for all dependent variables. All data collected was checked for normality through a Shapiro–Wilk test. Once normality was confirmed, parametric tests were employed with the significance value set at p ≤ 0.05. LT (W), $\dot{V}$O_2max_ (ml·kg·min^−1^) gross efficiency (%), peak ramp power (W), CP (W) and W’ (J) as well as perceptual responses (RPE) at LT and LT + 60 W were tested for between trial differences through a one‐way repeated measures ANOVA. In the presence of a significant effect (*p* ≤ 0.05), a Bonferroni‐corrected post hoc analysis was conducted for each dependant variable. Effect sizes were calculated as the mean difference between groups divided by the pooled standard deviation. Interpretation of effect size (Cohen's *d* (*d*)) was based on the guidelines provided by Hopkins et al. (2009), 0–0.19 trivial; 0.20–0.59 small; 0.6–1.19 moderate; 1.20–1.99 large and ≥ 2.00 very large. All statistics were generated using IBM SPSS (IBM Corp, Version 29.0. Armonk, NY).

## 3 Results

**FIGURE 2:**
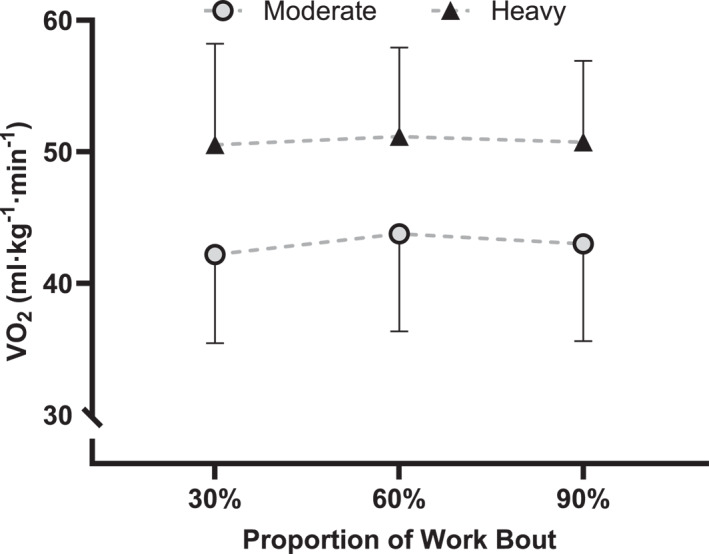
Mean ± SD $\dot{V}$O_2_ during the moderate and heavy 15 kJ⋅kg^−1^ work bouts.

Power output during the moderate and heavy 15 kJ⋅kg^−1^ (1099 ± 193 kJ) work bouts was 229.5 ± 41.4 W and 277.5 ± 50.9 W, with a duration of 4837 ± 675 s and 4000 ± 537 s, respectively. $\dot{V}$O_2_ was higher in the heavy bout (50.8 ± 6.8 mL⋅kg^−1^⋅min^−1^; *F* = 42.1 and *p* < 0.001) than the moderate bout (43 ± 7 mL⋅kg^−1^⋅min^−1^). $\dot{V}$O_2_ stabilised during the work bouts, such that there was no increase in $\dot{V}$O_2_ from 60% to 90% of the work duration (*p* = 0.76; see Figure 2).

**FIGURE 3:**
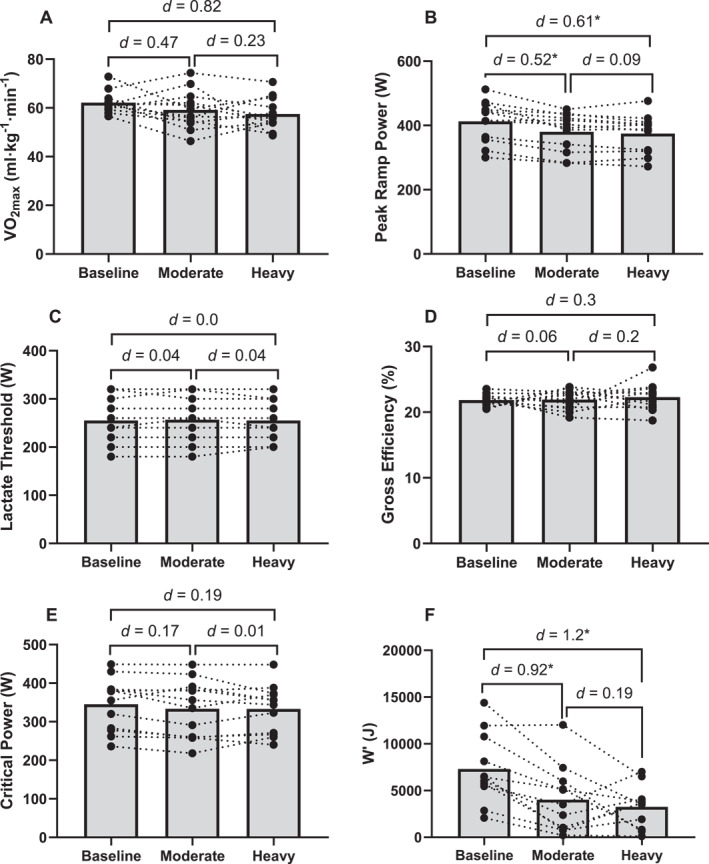
Changes in (A) $\dot{V}$O_2max_, (B) peak ramp power, (C) power at the lactate threshold, (D) gross efficiency, (E) critical power and (F) W’ (*n* = 11) after 15 kJ⋅kg^−1^ work in the moderate and heavy domain. Effect sizes (Cohen's *d*) between trials are annotated, with *p* < 0.05 denoted by *. Bars show the mean. Lines are individual participants.

There was no significant change in $\dot{V}$O_2max_ (*F* = 2.6 and *p* = 0.095; Figure 3A), power output at the lactate threshold (*F* = 0.12 and *p* = 0.86; Figure 3C) and gross efficiency (*F* = 0.3 and *p* = 0.74; Figure 3D) after the moderate and heavy work bouts. Peak ramp power significantly decreased (*F* = 28.3 and *p* = 0.001) after the moderate (*p* = 0.002 and *d* = 0.52) and heavy (*p* = 0.021 and *d* = 0.61) trial; peak ramp power was not significantly different between these trials though (*p* = 0.84 and *d* = 0.09; Figure 3B).

There was no significant change in critical power (*F* = 2.63 and *p* = 0.1; Figure 3E) after the work bouts. W′ was significantly reduced after completion of the moderate (*p* = 0.002 and *d* = 0.92) and heavy (*p* = 0.021 and *d* = 1.2) bouts; W′ was not different between moderate and heavy trials (*p* = 1 and *d* = 0.19; Figure 3F). RPE at the lactate threshold and 60 W above the lactate threshold was significantly elevated after the moderate and heavy work (*F* = 4.04 and *p* = 0.032).

## 4 Discussion

To our knowledge, this is the first controlled laboratory assessment of changes in $\dot{V}$O_2max_, lactate threshold, gross efficiency, CP and W′ after work‐matched bouts performed in the moderate and heavy exercise domain. The main findings are that: (a) only peak ramp power (the power obtained at the end of a graded exercise test to $\dot{V}$O_2max_) and W′ exhibited significant declines after 15 kJ·kg^−1^ of accumulated work and (b) the intensity of the prior work did not significantly affect the magnitude of this decline. This contradicts recent studies, which showed the intensity of prior work, rather than the volume, determined the presence (Barranco‐Gil et al. 2024; Mateo‐March et al. 2024) or magnitude (Spragg et al. 2024) of any decline in cyclists' power profiles.

Of all parameters associated with endurance performance, the durability of CP has received the most attention. In our study, although CP was 3.5%–3.7% lower after 15 kJ·kg^−1^ of moderate or heavy exercise, these differences were not significant and were trivial in magnitude (*d* = 0.17–0.19). In contrast, initial studies on durability showed a significant ∼8% decline in CP after 2 h heavy cycling (Clark et al. 2018, 2019b). One plausible explanation for this discrepancy is the shorter exercise duration used here (120 min vs. 66–80 min). This duration is less likely to deplete participants' endogenous carbohydrate stores, which is thought to be a key determinant of durability (Clark et al. 2019b). Indeed, Clark et al. (2019b) found that CP only decreased after 2 h, but not after 40 and 80 min, of heavy exercise. More recently, Barranco‐Gil et al. (2024) observed no significant decline in CP after 15 kJ·kg^−1^ (equivalent to the current study) bouts performed above or below the CP in competitive junior cyclists. Taken together, this would indicate that work and duration mediate changes in CP, with prolonged exercise bouts likely to be more deleterious.

Recent investigations have suggested that the intensity of exercise, rather than work or duration, is the primary determinant of alterations in endurance parameters. Spragg et al. (2024) observed greater reductions in mean maximal power during 15 s and 3 min (but not 12 min) work bouts after 5 × 8 min bouts at 105%–110% CP compared to more work (∼2000 kJ) at a moderate intensity (< 70% CP). Similarly, Barranco‐Gil et al. (2024) reported reductions in mean maximal power during a 2 min test (but not CP and mean maximal power during 5 and 12 min tests) after 15 kJ·kg^−1^ of work involving 3 min repetitions at 110%–120% CP but not when participants performed the same amount of work at a moderate intensity. Finally, Mateo‐March et al. (2024) showed that mean maximal power during 5 s, 5 min, 10 min and 15 min tests, alongside CP, all declined after as little as 2.5 kJ·kg^−1^ of work performed above CP but not when the same work was performed below CP. These findings contrast our own observation that there was no difference in any endurance parameter between moderate and heavy trials. This is surprising given that determinants of fatigue, and therefore presumably durability, are to some extent dependent on the exercise domain (Burnley and Jones 2007). However, it should be noted that all studies that have explored the effect of exercise intensity on durability have performed work above the CP and therefore in the severe, not heavy, exercise domain. Exercise in this domain is associated with greater peripheral disturbances associated with high‐energy phosphate depletion and fatiguing metabolites (e.g., H^+^ and P_i_) that can accumulate rapidly (Black et al. 2017; Jones et al. 2008)—something that might explain why deteriorations in mean maximal power are typically seen during shorter and more intense exercise tests that are likely to be affected by such disturbances (Barranco‐Gil et al. 2024; Spragg et al. 2024). In contrast, fatigue in the heavy exercise domain is likely to be associated with factors such as glycogen depletion and hyperthermia, which can take several hours to occur (Hawley et al. 1997). As such, although durability might be affected by the domain of prior exercise, it is likely that exercise in the heavy domain needs to be of sufficient duration (longer than 60–80 min) for this to manifest.

It is notable that we only observed significant reductions in peak ramp power and W′ after either of the exercise bouts, albeit there were also small‐to‐moderate (*d* = 0.5–0.8) nonsignificant effects on $\dot{V}$O_2max_. We are not aware of any other studies which have assessed durability of $\dot{V}$O_2max_, but our observation seems consistent with Clark et al.'s (2018, 2019a), that peak $\dot{V}$O_2_ in a 3 min all out test did not change after 2 h of heavy exercise. As such, observed performance decrements associated with moderate or heavy exercise do not appear to be due to changes in $\dot{V}$O_2max_. The significant 7%–9% change in peak ramp power in this study is consistent with prior observations of a decline in power during performance tests of different durations (Barranco‐Gil et al. 2024; Mateo‐March et al. 2022, 2024; Spragg et al. 2024). Similarly, Clark et al. (2018, 2019a, 2019b) and Barranco‐Gil et al. (2024) reported reductions in W′ of 20%–30% after heavy and severe exercise. The precise mechanism(s) for the decline in these parameters is not clear and requires further research. A decline in high‐energy phosphates and accumulation of peripheral metabolites, particularly in type II muscle fibres, would likely reduce peak ramp power and W’; however, this is unlikely to have occurred at the moderate and heavy exercise intensities used here (Black et al. 2017; Jones et al. 2008). As previously discussed, glycogen depletion has been posited to affect CP and W′, but we feel that the duration of exercise (particularly in the heavy domain) was unlikely to have depleted glycogen sufficiently—it is possible that there was localised glycogen depletion in certain fibres that affected peak ramp power and W′ though (Nielsen et al. 2011). Another possibility is that these parameters were affected by central fatigue, which is more prominent during exercise performed below the CP (Black et al. 2017; Burnley et al. 2012). Indeed, during a critical torque test requiring maximal voluntary contractions, maximal torque and voluntary activation of motor units decline concurrently until critical torque is reached (Burnley 2009).

We observed no significant change in lactate threshold (demarcating the transition from moderate to heavy exercise) or gross efficiency after moderate or heavy exercise bouts. This contrasts the recent work of Stevenson et al. (2022), who found 2 hours of cycling at 90% of the ventilatory threshold resulted in a ∼10% decline in the power at ventilatory threshold, ascribed to a reduction in metabolic power and loss of energetic efficiency due to progressive recruitment of type II fibres, muscle glycogen depletion and increased fat oxidation. It is possible that the longer exercise duration used by Stevenson et al. (2022) is necessary to decrease metabolic power and gross efficiency, resulting in a reduced power at the moderate‐to‐heavy exercise intensity threshold. Contrasting this notion, Passfield and Doust (2000) showed that only 60 min of cycling at ∼60%VO_2peak_ significantly reduced gross efficiency by 1.8%. Recent research by Gallo et al. (2024) might explain these seemingly contradictory findings. They reported that the gas exchange threshold did not change after 1 h of moderate intensity cycling but decreased after 2–5 h. Importantly, the decline was nonlinear, highly individual and only present prior to task failure. Similar to the work of Stevenson et al. (2022), this decline was ascribed to reduced energy expenditure and gross efficiency related to increased fat oxidation. Therefore, the durability of gross efficiency, and subsequent power at the moderate‐to‐heavy transition, is likely duration‐dependent in that an individual needs to exercise long enough for fat oxidation to increase and gross efficiency to decline—something that will be dependent on the individual. This might also explain why Pro Team cyclists exhibit greater decays in mean maximal power after prior work bouts than World Tour cyclists (Mateo‐March et al. 2022). Finally, it should not be discounted that methodological differences and statistical power between studies could explain discrepancies. Further exploration of the determinants of durability of the lactate threshold and gross efficiency are therefore warranted.

Although our sample size is in keeping with other studies in the area and we met the requirements of an a priori sample size estimation, it is possible that we lacked statistical power to detect effects in all our variables (where differences were typically small)—therefore, this might be considered a limitation of our work. The low W′ of our participants (∼8 kJ), compared to those reported elsewhere (12–18 kJ), also warrants comment. We do not believe this is due to the fitness of our participants, as the $\dot{V}$O_2max's_ reported here (62.1 mL·kg^−1^ min^−1^) are in keeping, and often higher, than those of participants in similar studies (∼52–71 mL·kg^−1^ min^−1^). Instead, we believe this is due to the protocol employed to determine CP and W’. Caen et al. (2023) showed that although CP attained from a ramp test and constant work‐rate trials are comparable, the W′ from two protocols should not be used interchangeably owing to a lower W’. A plausible explanation for constant work‐rate trials producing larger W′ values is that longer trials are subject to a greater influence of motivation curtailing the longer constant work rate trials, which would lower CP and elevate W' (Morton et al. 1997). Additionally, the participants we recruited were mainly time trial specialists, meaning that their training would naturally be manipulated to increase CP more than W′ to suit the demands of their races.

To conclude, this study shows that peak ramp power and W′ decline after 15 kJ·kg^−1^ of cycling, whereas critical power, gross efficiency and the lactate threshold were not significantly changed. For the first time, we also show that when the quantity of work is matched, moderate and heavy work bouts exhibit similar durability responses, at least for the duration of exercise explored here (∼60–80 min). Exercise physiologists, coaches and athletes should be conscious of the shifts in some physiological parameters after relatively small workloads (15 kJ·kg^−1^) as the magnitude of these downward shifts, or durability, is likely to influence endurance performance. Further research is now needed to explore durability after exercise in different domains of varying duration and amounts of work. Research exploring the physiological mechanisms for observed effects is also warranted.

## Ethics Statement

This research was granted institutional ethics approval before data collection.

## Consent

All participants provided written informed consent before their participation.

## Conflicts of Interest

The authors declare no conflicts of interest.
